# Betacellulin regulates peripheral nerve regeneration by affecting Schwann cell migration and axon elongation

**DOI:** 10.1186/s10020-021-00292-5

**Published:** 2021-03-25

**Authors:** Yaxian Wang, Fuchao Zhang, Yunsong Zhang, Qi Shan, Wei Liu, Fengyuan Zhang, Feiyu Zhang, Sheng Yi

**Affiliations:** 1 Key Laboratory of Neuroregeneration of Jiangsu and Ministry of Education, Co-Innovation Center of Neuroregeneration, NMPA Key Laboratory for Research and Evaluation of Tissue Engineering Technology Products, Nantong University, Nantong, 226001 Jiangsu China; 2 Medical School of Nantong University, Nantong, 226001 Jiangsu China

**Keywords:** Peripheral nerve injury, Betacellulin, Schwann cell migration, Axon elongation, Nerve regeneration

## Abstract

**Background:**

Growth factors execute essential biological functions and affect various physiological and pathological processes, including peripheral nerve repair and regeneration. Our previous sequencing data showed that the mRNA coding for betacellulin (Btc), an epidermal growth factor protein family member, was up-regulated in rat sciatic nerve segment after nerve injury, implying the potential involvement of Btc during peripheral nerve regeneration.

**Methods:**

Expression of Btc was examined in Schwann cells by immunostaining. The function of Btc in regulating Schwann cells was investigated by transfecting cultured cells with siRNA segment against *Btc* or treating cells with Btc recombinant protein. The influence of Schwann cell-secreted Btc on neurons was determined using a co-culture assay. The in vivo effects of Btc on Schwann cell migration and axon elongation after rat sciatic nerve injury were further evaluated.

**Results:**

Immunostaining images and ELISA outcomes indicated that Btc was present in and secreted by Schwann cells. Transwell migration and wound healing observations showed that transfection with siRNA against *Btc* impeded Schwann cell migration while application of exogenous Btc advanced Schwann cell migration. Besides the regulating effect on Schwann cell phenotype, Btc secreted by Schwann cells influenced neuron behavior and increased neurite length. In vivo evidence supported the promoting role of Btc in nerve regeneration after both rat sciatic nerve crush injury and transection injury.

**Conclusion:**

Our findings demonstrate the essential roles of Btc on Schwann cell migration and axon elongation and imply the potential application of Btc as a regenerative strategy for treating peripheral nerve injury.

**Supplementary Information:**

The online version contains supplementary material available at 10.1186/s10020-021-00292-5.

## Introduction

Although peripheral nerves exhibited greater regenerative abilities after nerve injury as compared with central nerves, peripheral nerve injury, especially peripheral nerve injury with long nerve defects, is still a severe health problem (Aguilar 2012). The success of peripheral nerve regeneration relies on the activation of neuronal intrinsic growth capacity and the construction of a permissive microenvironment (Chen et al. 2007). Local administration of proteins and molecules with neurotrophic and neuroregenerative properties largely benefits nerve regeneration and is considered as a promising regenerative strategy (Tajdaran et al. 2019).

Growth factors are natural molecules that function to regulate cellular behaviors. Numerous growth factors, such as nerve growth factor, brain-derived neurotrophic factor, glial cell line-derived neurotrophic factor, and fibroblast growth factors have been identified as essential neurotrophic factors that benefit nerve repair (Gu et al. 2011, 2014). Our recent study screened critical growth factors that were differentially expressed after rat sciatic nerve injury by the joint use of high-throughput sequencing data and advanced bioinformatic tools and found that besides these well-investigated growth factors, *Btc*, a gene encoding for betacellulin (Btc), was robustly up-regulated in the injured sciatic nerve segments (Zhang et al. 2019).

Btc is a secreted growth factor that belongs to the epidermal growth factor protein family. Btc has been identified as a potent mitogen for different types of cells, including retinal pigment epithelial cells, vascular smooth muscle cells, β cells, retinal progenitor cells, and neural stem cells (Huotari et al. 1998; Shing et al. 1993; Zhang et al. 2018; Oh et al. 2011; Gomez-Gaviro et al. 2012). A recent study demonstrated that Btc could also drive the proliferation of Schwann cells, a unique type of glial cells in the peripheral nervous system (Vallieres et al. 2017). Notably, besides proliferation and myelination, the migration of Schwann cells is also essential for successful peripheral nerve repair as migrating Schwann cells form a cord in the nerve bridge, generate a regenerative microenviroment, and guide axon regeneration (Chen et al. 2019; Clements et al. 2017). The functions of Btc on Schwann cell migration, however, have not yet been investigated.

To gain a more comprehensive understanding of the biological roles of growth factor Btc during peripheral nerve regeneration, we cultured primary Schwann cells, transfected Schwann cells with siRNA segment against *Btc* or treated Schwann cells with Btc recombinant protein, and performed Transwell migration assay and wound healing assay to observe the effect of Btc on Schwann cell migration. Considering that Btc is a secreted protein, the biological functions of Schwann cell-secreted Btc on neurons were determined. The in vivo effect of Btc on Schwann cell migration and axon elongation after rat sciatic nerve injury were further evaluated.

## Materials and methods

### Ethical statement

Sprague–Dawley (SD) rats were obtained from the Experimental Animal Center of Nantong University. Animal experiments were conducted based on Institutional Animal Care Guidelines of Nantong University and approved ethically by the Administration Committee of Experimental Animals, Jiangsu, China.

### Schwann cell culture and treatment

Primary Schwann cells were collected and cultured as previously described (Yi et al. 2019a). Briefly, cells isolated from neonatal SD rat sciatic nerves were purified with anti-Thy1.1 antibody (Sigma, St. Louis, MO, USA) and rabbit complement (Invitrogen, Carlsbad, CA, USA) and cultured in DMEM (Gibco, Grand Island, NY, USA) containing 10% FBS (Gibco), 1% penicillin and streptomycin (Invitrogen), 2 μM forskolin (Sigma), and 10 ng/ml HRG (R&D Systems Inc., Minneapolis, MN, USA). For *Btc* knockdown, Schwann cells were transfected with siRNA segments targeting *Btc* (siRNA sequences: siRNA-1: TCTTCGGAAACATCGCAAA, siRNA-2: CAAGCATTACTGCATCCAT, and siRNA-3: GAAACCAATGGCTCTCTTT) or a non-targeting negative control (random sequence, RiboBio, Guangzhou, Guangdong, China) for 36–48 h using Lipofectamine RNAiMAX transfection reagent (Invitrogen). For Btc protein exposure, Schwann cells were treated with 10 ng/ml Btc recombinant protein (100–50-20, PeproTech, Rocky Hill, NJ, USA) dissolved in 0.1% BSA or 0.1% BSA control for 24 h.

### Immunofluorescence staining

Cultured Schwann cells were fixed with 4% paraformaldehyde and exposed to primary antibodies rabbit anti-Btc (1:100, PA5-76664, Invitrogen) and mouse anti-S100 (1:100, ab4066, Abcam, Cambridge, MA, USA) followed by reaction with secondary antibodies Alexa Fluor 488-conjugated anti-rabbit (1:400, ab150077, Abcam) and Cy3-conjugated anti-Mouse (1:400, SA00009-1, Proteintech, Rosemont, IL). Cell nuclei were stained with DAPI Fluoromount-G (Southern Biotech, Birmingham, AL, USA). Images were captured using Zeiss Axio Imager M2 (Carl Zeiss Microscopy GmbH, Jena, Germany).

### Real-time RT-PCR

RNA samples were isolated from cultured Schwann cells using RNA-Quick Purification Kit (YiShan Biotechnology Co. LTD, Shanghai, China) according to the manufacturer’s instructions. Briefly, Schwann cells were washed, lysed with Lysis Buffer, and mixed with equal volume ethanol. Liquid was added to the spin column, centrifuged, and washed. RNA samples were collected using Elution Buffer. The concentrations of isolated RNA samples were determined using NanoDrop. 1 μg RNA sample was reverse transcripted to cDNA with a concentration of 0.05 μg/μl. cDNA was diluted to 5 ng/μl to perform PCR experiment using SYBR Green Premix Ex Taq (TaKaRa) on a StepOne Real-time PCR System (Applied Biosystems, Foster City, CA, USA). The relative mRNA expression of *Btc* was measured using the 2^−ΔΔCt^ method. Primer sequences were: *Btc* (forward) 5′-TCTCCAGTGCGTGGTGG-3′ and (reverse) 5′-CGAGAGAAGTGGGTTTTCGATT-3′ and *Gapdh* (forward) 5′- ACAGCAACAGGGTGGTGGAC-3′ and (reverse) 5′- TTTGAGGGTGCAGCGAACTT-3’.

### ELISA

A total of 6 × 10^4^ Schwann cells transfected with *Btc* siRNA or siRNA control, respectively, were cultured in DMEM for additionally 24 h. Schwann cell culture supernatants were collected and passed through a 0.22 μm filter (Millipore, Billerica, MA, USA). The amount of secreted Btc protein was determined with a Btc ELISA Kit (ARG81876, Arigo biolaboratories, Hsinchu, Taiwan, China) according to manufacturer’s instructions using a Synergy™ 2 Multi-Mode Microplate Reader (BioTek, Burlington, VT, USA).

### Transwell migration assay

To determine the effect of Btc on Schwann cell migration, Schwann cells transfected with *Btc* siRNA or siRNA control were suspended in DMEM and transferred to the upper chamber of a Transwell with 8 μm pores (Costar, Cambridge, MA, USA). The bottom chamber of the Transwell was filled with cell culture medium with 10% FBS to drive cell migration. In addition, cultured primary Schwann cells were directly seeded onto the upper chamber. Btc recombinant protein was added to both the upper and bottom chambers to reach a concentration of 10 ng/ml. In both cases, the upper surface of the upper chamber was cleaned after 24 h and the bottom surface of the upper chamber was stained with 0.1% crystal violet. Stained crystal violet was dissolved with 33% acetic acid to measure absorbance using a Synergy™ 2 Multi-Mode Microplate Reader (BioTek). Images were captured using a DMI 3000B inverted microscope (Leica Microsystems, Bensheim, Germany).

### Wound healing assay

Schwann cells transfected with *Btc* siRNA or siRNA control or exposed to Btc recombinant protein were suspended in cell culture medium at a density of 2 × 10^4^ cells/ml and transferred to a mold chamber with a 1 mm wide insert. Insert was removed after cell reaching > 95% confluent to allow cell migration. Remaining cleaned areas were captured using a DMI 3000B inverted microscope (Leica Microsystems) 9 h later. Cleaned areas in each image were measured to determine percentage decrease in scratch using Image-Pro Plus (Media Cybernetics, Rockville, MD, USA).

### Neuron culture

Briefly, dorsal root ganglia were obtained from neonatal SD rats, cut into pieces, and digested with collagenase I. Isolated cells were re-suspended in 15% BSA and subjected to centrifuge to collect pellets. Collected primary neurons were cultured in Neurobasal medium (Gibco) containing 2% B27 supplement (Gibco), 2 mM l-glutamine (ThermoFisher Scientific, Waltham, MA, USA), and 1% penicillin and streptomycin (Invitrogen) in a humidified 5% CO_2_ incubator 37 °C.

Neurons were co-cultured with Schwann cells transfected with *Btc* siRNA or siRNA control using Transwell co-culture assay. Transfected Schwann cells were suspended in DMEM and seeded to the upper chamber of a Transwell with 8 μm pores. Neurons were suspended in neuron culture medium and seeded to a PLL-coated glass slide placed on the bottom chamber. No Schwann cells were observed to be migrated to the bottom chamber. After 24-h co-culture, neurons were fixed with 4% paraformaldehyde and immunostained with primary antibody mouse anti-neurofilament-200kD (NF-H; 1:200, N2912, Sigma) followed by secondary antibody Cy3-conjugated Goat anti-Mouse IgG (1:400, SA00009-1, Proteintech). Cell nuclei were stained with DAPI Fluoromount-G (Southern Biotech, Birmingham, AL, USA). Images were captured using Zeiss Axio Imager M2 (Carl Zeiss Microscopy GmbH). Total neurite length was determined using Image J (National Institutes of Health, Bethesda, MA, USA).

### In vivo experiments

Adult male SD rats weighting 180–220 g were subjected sciatic nerve crush and Btc recombinant protein injection to evaluate the in vivo effects of Btc. After anaesthetization, sciatic nerves of SD rats were exposed and crushed with a forcep. After sciatic nerve crush injury, 100 ng Btc recombinant protein was dissolved in 0.1% BSA and diluted in 5 µl saline. Btc or equal volume of saline was injected into the epineurium at the crush site using a microsyringe. Rat sciatic nerve segments were collected at 4 days after crush injury, fixed with 4% paraformaldehyde, cut with a cryostat (Leica Microsystems, Bensheim, Germany) into 12 µm thick nerve sections, immunostained with primary antibodies rabbit anti-SCG10 (1:500, NBP1-49461, Novus Biologicals, Littleton, CO, USA) and mouse anti-neurofilament-200kD (NF-H; 1:200, N2912, Sigma) overnight at 4 °C, and exposed to secondary antibodies Alexa Fluor 488-conjugated anti-mouse secondary antibody (1:400, ab150113, Abcam, Cambridge, MA, USA) and Alexa Fluor 555-conjugated anti-rabbit secondary antibody (1:400, ab150078, Abcam) for 2 h at room temperature. Images were captured using Zeiss Axio Imager M2 (Carl Zeiss Microscopy GmbH).

Another group of SD rats were subjected sciatic nerve transection to generate a 6 mm nerve gap. A silicone tube filled with 100 ng Btc recombinant protein dissolved in 0.1% BSA and 8 µl saline or equal volume of saline was implanted to bridge the nerve gap. Rat sciatic nerve segments were collected at 10 days after transection and immunostained with primary antibodies mouse anti-NF-H and rabbit anti-S100β (1:200, S2644, Sigma) and secondary antibodies Alexa Fluor 488-conjugated anti-mouse secondary antibody and Alexa Fluor 555-conjugated anti-rabbit secondary antibody. Cell nucleus was stained with Hoechst 33342 (ab228551, Abcam). Images were captured using Zeiss Axio Imager M2 (Carl Zeiss Microscopy GmbH). Axon elongation length and Schwann cell migration distance were calculated using Zeiss Microscopy Software ZEN 2.3.

### Statistic analysis

Statistical analysis and graphs were conducted using GraphPad Prism 6.0 (GraphPad Software, Inc., La Jolla, CA, USA). Numerical data were displayed in scatter plot as median with range. A p-value < 0.05 was considered as significantly different.

## Results

### Btc stimulated Schwann cell migration

**Fig. 1:**
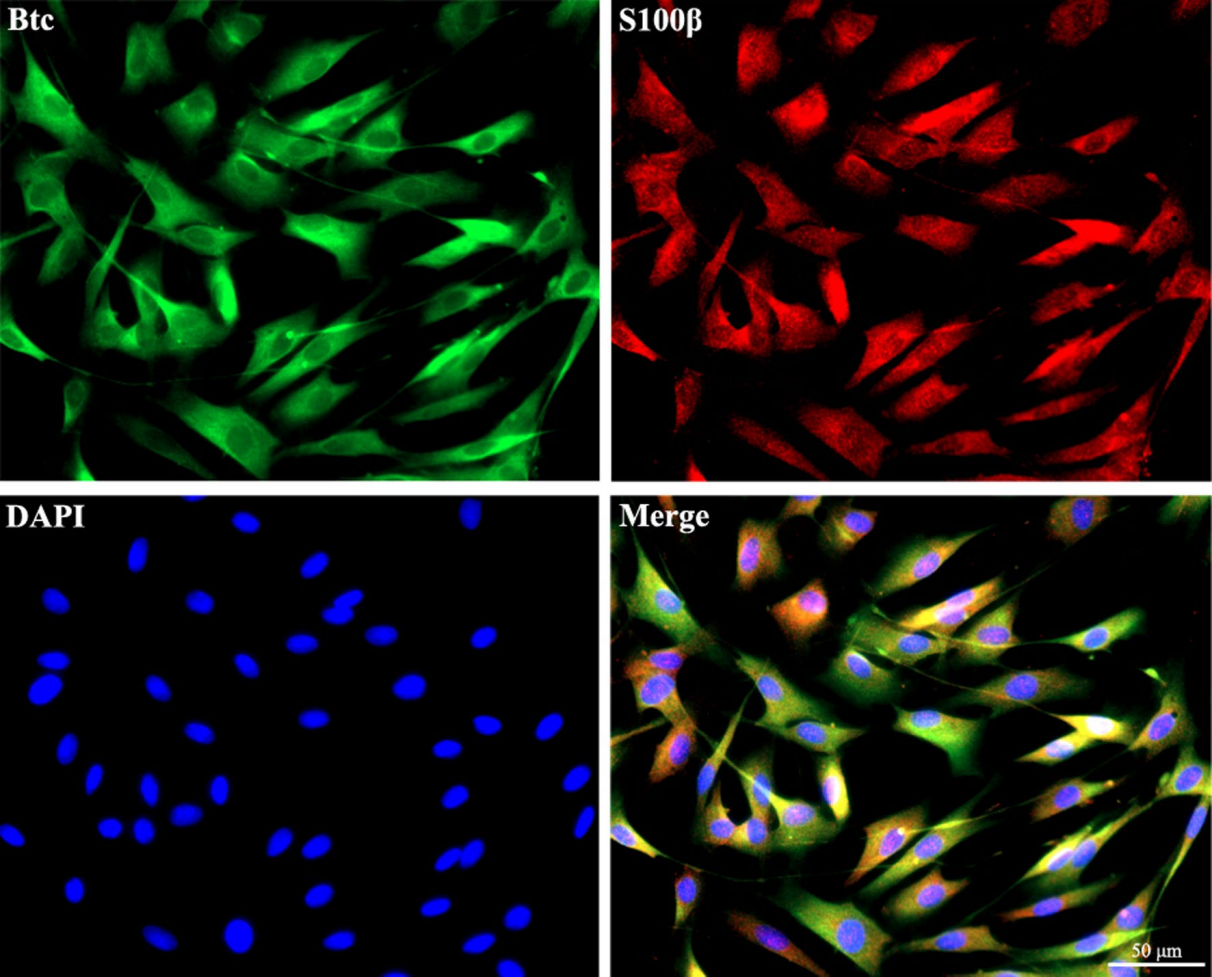
Immunostaining of Btc in cultured Schwann cells. Red color indicated S100β, green color indicated Btc, and blue color indicated nucleus. Scale bars indicated 50 µm

Isolated and cultured Schwann cells were immunostained with Schwann cell marker S100β to assess cell purity. Immunofluorescence images showed that the ratio of the number of S100β-positive cells to that of DAPI-positive cells was > 95%, indicating that we achieved Schwann cells with high purity. Further immunostaining of cultured Schwann cells with Btc showed that the majority of Btc-positive signals overlapped with S100β-positive signals, suggesting the expression and existence of Btc protein in Schwann cells (Fig. 1).

**Fig. 2:**
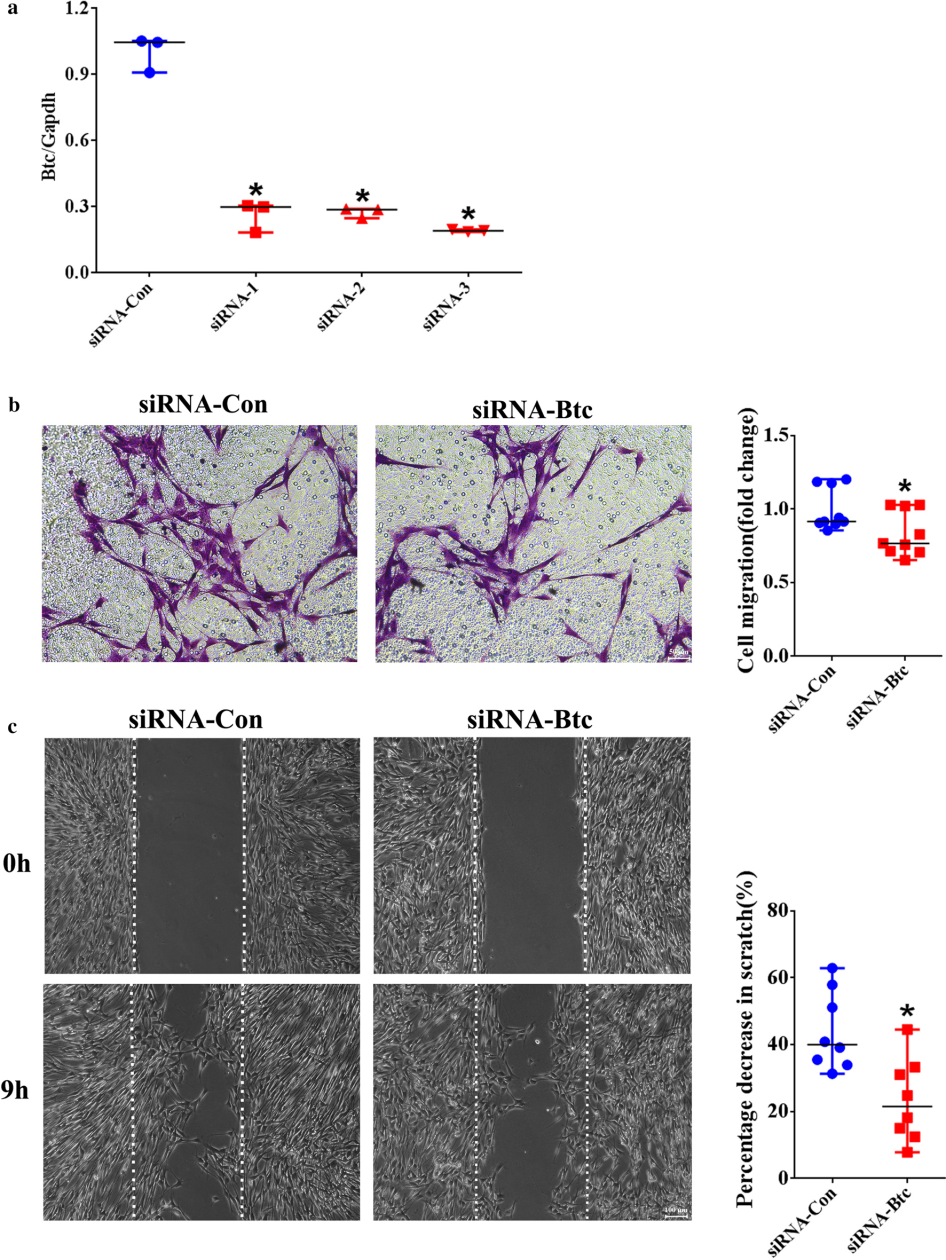
The in vitro effect of siRNA against *Btc* on Schwann cell migration. **a** The transfection efficiency of siRNA-*Btc*. Relative abundance of *Btc* was summarized from three experiments. Statistical evaluation was performed using ANOVA. *p-value < 0.05 versus siRNA control. **b** Representative Transwell migration images and normalized averaged migration rate of Schwann cells transfected with siRNA control or siRNA-*Btc*. Scale bars represented 50 μm. Transwell migration assay was done three times using duplicate or triplicate wells. Statistical evaluation was performed using Mann–Whitney test. *p-value < 0.05 versus siRNA control. **c** Representative wound healing images and percentage decrease in scratch of Schwann cells transfected with siRNA control or siRNA-*Btc*. Scale bars represented 100 μm. Transwell migration assay was done four times using duplicate wells. Statistical evaluation was performed using Mann–Whitney test. *p-value < 0.05 versus siRNA control

Schwann cells were then transfected with siRNA against *Btc* to evaluate the functional effects of reduced *Btc* expression. Transfection of Schwann cells with three siRNA segments against *Btc* (designated as siRNA-1, siRNA-2, and siRNA-3) all successfully decreased the mRNA abundance of *Btc* (Fig. 2a). Segment siRNA-3 achieved a relatively high and stable silencing efficiency and was subsequently used for *Btc* knockdown. Following transfection, Schwann cells were seeded onto the upper chamber of a Transwell to observe cellular migration ability. Compared with cell transfected with siRNA control, less Schwann cells were observed to be migrated across the upper surface of Transwell upper chamber (Fig. 2b). The migration ability of Schwann cells was further examined by generating an equal wide blank space and measuring the area of left blank space at 9 h after cell culture. Remaining cleaned area was much larger in Schwann cells transfected with *Btc* siRNA as compared with cells transfecting with siRNA control. Summarized data showed that Schwann cells transfected with *Btc* siRNA only had about 20% of area decrease in scratch while Schwann cells transfected with siRNA control had nearly twofold of area decrease in scratch (Fig. 2c).

**Fig. 3:**
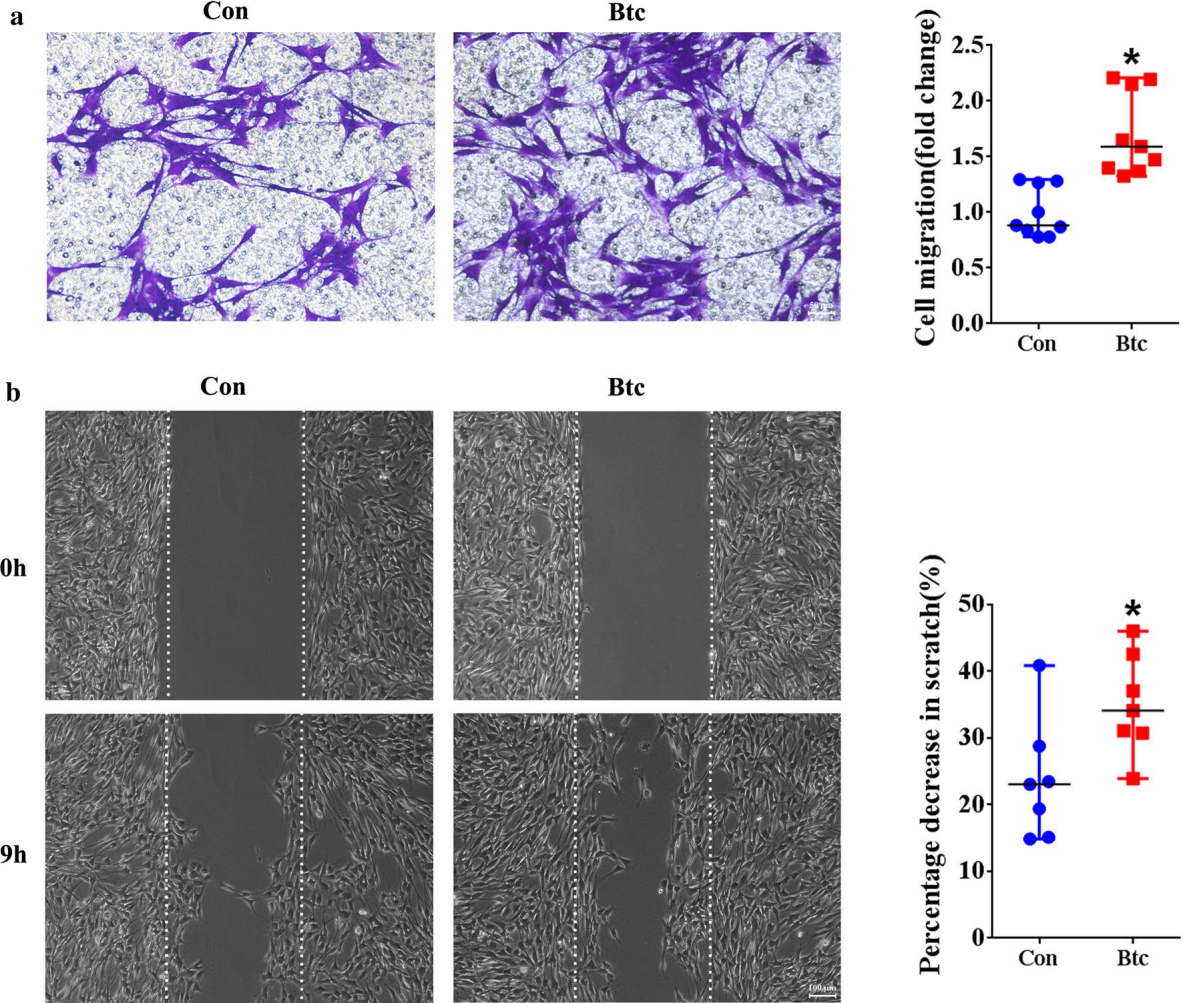
The in vitro effect of Btc recombinant protein on Schwann cell migration. **a** Representative Transwell migration images and normalized averaged migration rate of Schwann cells treated with or without Btc recombinant protein. Scale bars represented 50 μm. Transwell migration assay was done three times using duplicate or triplicate wells. Statistical evaluation was performed using Mann–Whitney test. *p-value < 0.05 versus non-treated cells. **b** Representative wound healing images and percentage decrease in scratch of Schwann cells treated with or without Btc recombinant protein. Scale bars represented 100 μm. Transwell migration assay was done three times using duplicate or triplicate wells. Statistical evaluation was performed using Mann–Whitney test. *p-value < 0.05 versus non-treated cells

Besides cell transfection, Schwann cells were also directly exposed to Btc recombinant protein. On the contrary to cells transfected with *Btc* siRNA, following Btc recombinant protein treatment, the amount of migrated Schwann cells was increased (Fig. 3a) while percentage decrease of the cleaned area in scratch was elevated as well (Fig. 3b). These observations indicate that Btc promote the migration of Schwann cells.

### Schwann cell-secreted Btc stimulated axon elongation

**Fig. 4:**
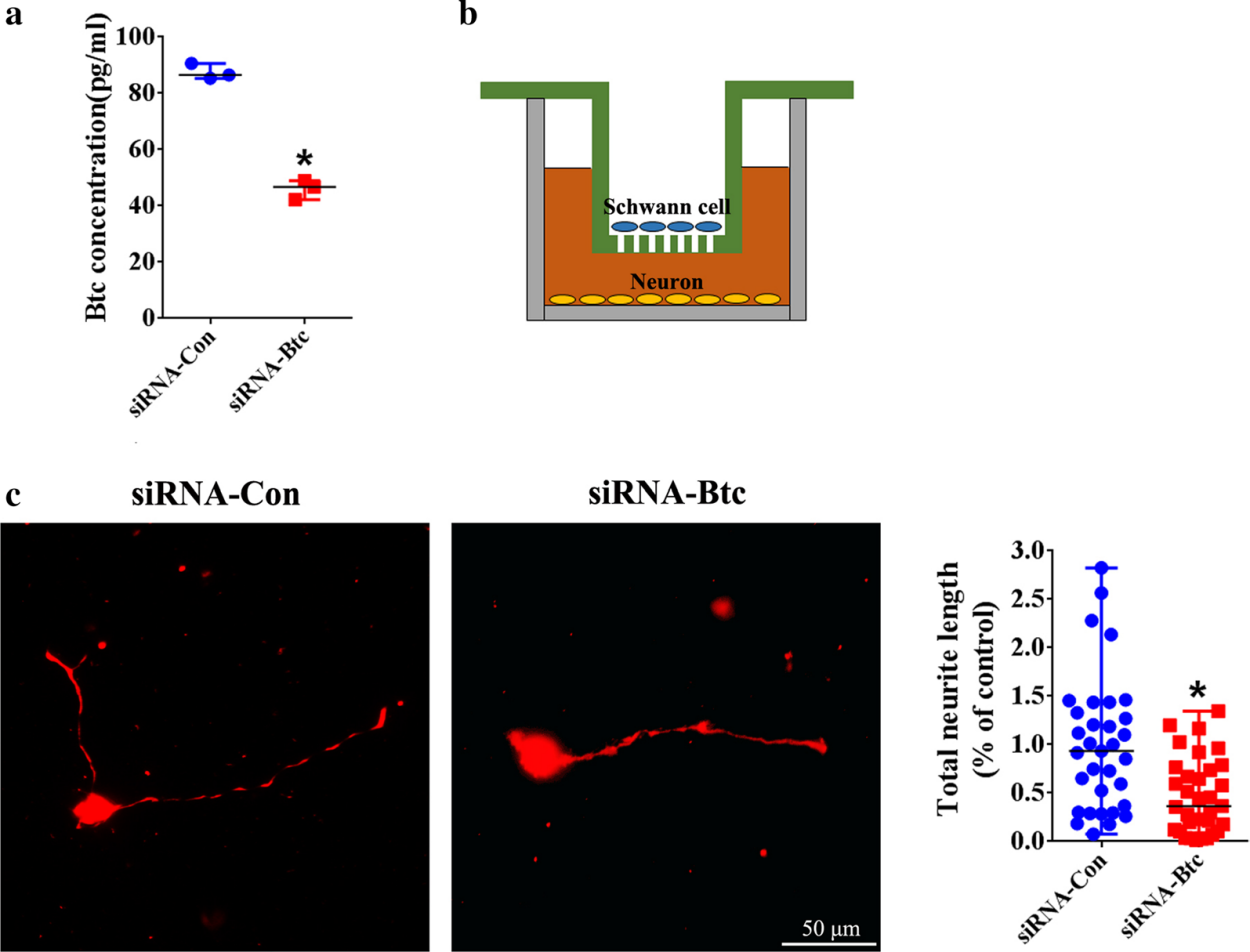
The in vitro effect of Schwann cell-secreted Btc on axon elongation. **a** The amount of Schwann cell-secreted Btc in Schwann cells transfected with siRNA control or siRNA-*Btc*. The concentration of *Btc* was summarized from three experiments. Statistical evaluation was performed using t-test. *p-value < 0.05 versus siRNA control. **b** The schematic diagram of the co-culture of Schwann cells and neurons. **c** Representative immunofluorescence images and normalized neurite length of neurons co-cultured with transfected with siRNA control or siRNA-*Btc*. Scale bars represented 50 μm. Neurite length was summarized from three experiments. Statistical evaluation was performed using Mann–Whitney test. *p-value < 0.05 versus neurons co-cultured with Schwann cells transfected with siRNA control

ELISA was performed to determine the amount of Btc in the supernatant of cultured Schwann cells. ELISA readings revealed that certain level of Btc protein could be detected in Schwann cell supernatant while transfection of Schwann cells with *Btc* siRNA largely reduced the secretion of Btc protein (Fig. 4a).

Schwann cells were then in-directly co-cultured with neurons to examine the effect of secreted Btc protein on neuron behaviors (Fig. 4b). Neurons co-cultured with Schwann cells transfected with siRNA control possessed much longer neurites as compared with neurons co-cultured with Schwann cells transfected with siRNA against *Btc* (Fig. 4c). It implies that Schwann cell-secreted Btc stimulate the neurite elongation.

### Btc promoted the repair of injured peripheral nerves

**Fig. 5:**
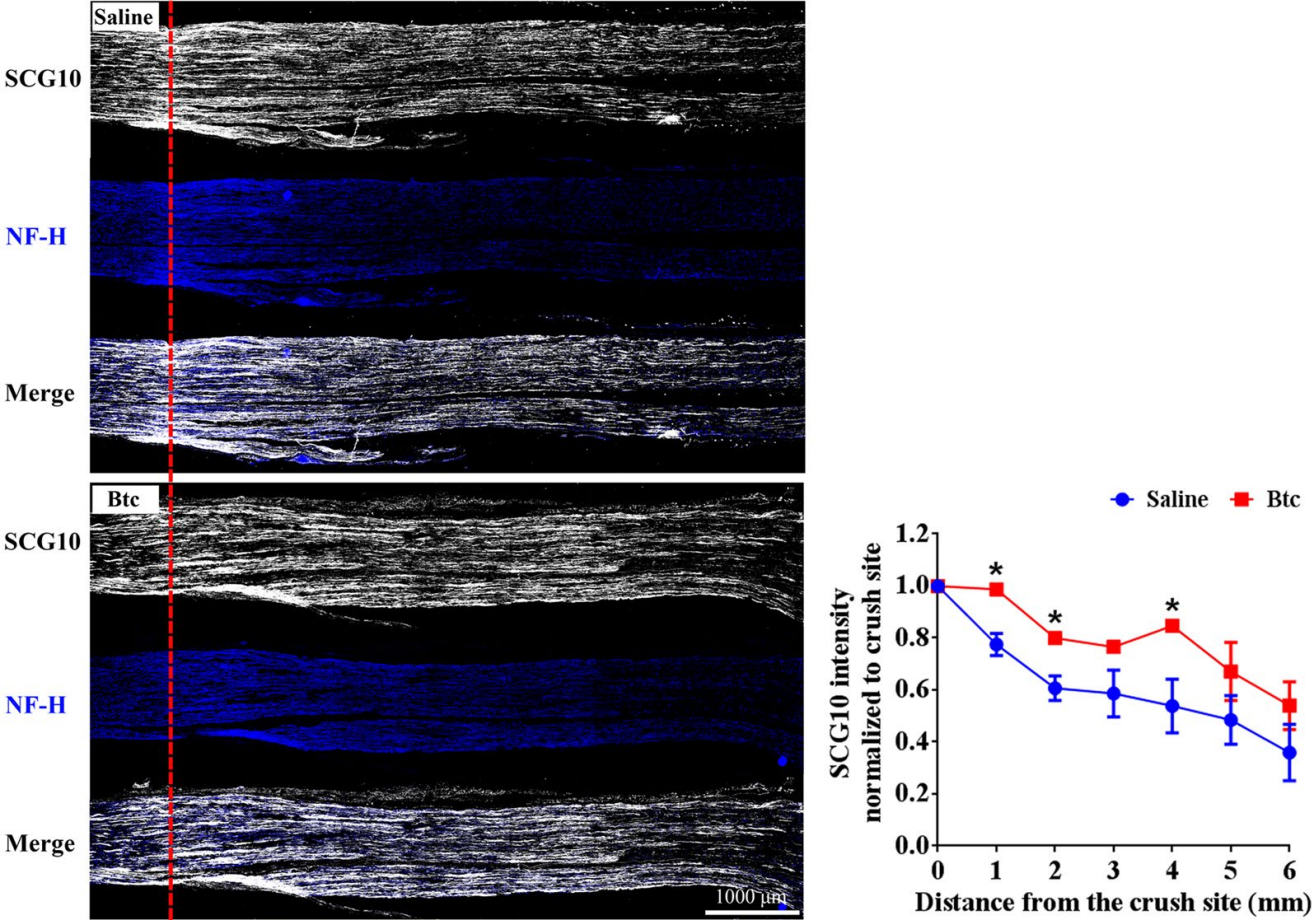
The in vivo effect of Btc recombinant protein after rat sciatic nerve crush. Representative immunofluorescence images of rat sciatic nerve segments treated with saline control or Btc recombinant protein at 4 days after nerve crush. Averaged intensity of SCG10 at 1, 2, 3, 4, 5, and 6 mm from the crush site were normalized to its intensity at the crush site. Scale bar indicated 1000 µm. Axon length was summarized from three experiments. Statistical evaluation was performed using multiple t-test. *p-value < 0.05 versus saline control

In addition to in vitro examinations, Btc recombinant protein was directly applied to SD rats to observe the in vivo effect of Btc after peripheral nerve injury. First, rat sciatic nerves subjected crush injury were injected with Btc recombinant protein or saline control. Injured sciatic nerve segments were collected at 4 days after nerve crush injury and immunostained with axon markers SCG10 and NF-H. In both Btc recombinant protein-treated group and saline-treated group, regenerated axons grow across the crushed site towards their target organs. However, the intensity of SCG10 seemed to be higher at multiple measured positions in Btc recombinant protein-treated group, especially at 1, 2, and 4 mm from the crush site (Fig. 5).

**Fig. 6:**
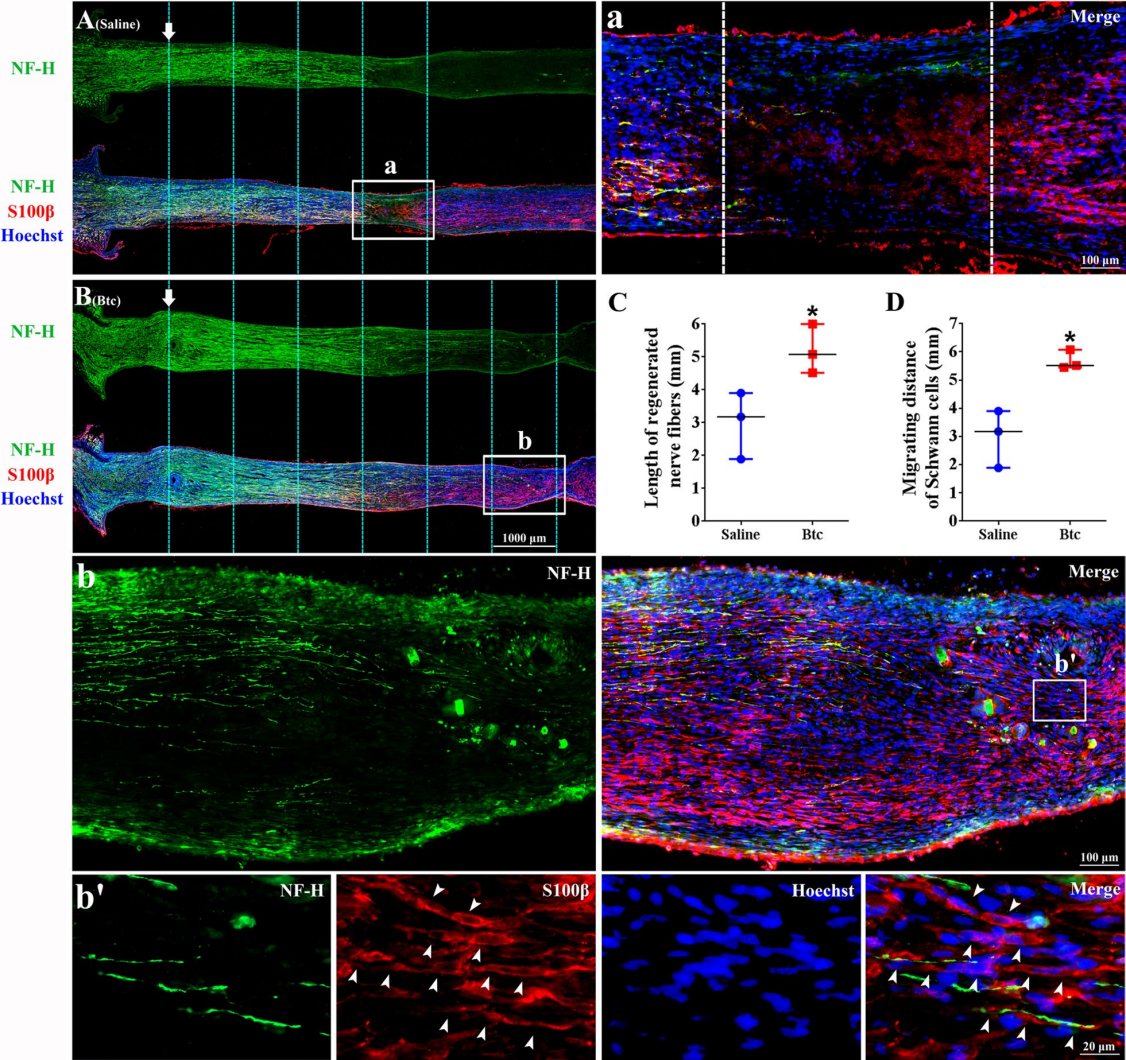
The in vivo effect of Btc recombinant protein after rat sciatic nerve transection and silicone bridging. Representative immunofluorescence images of rat sciatic nerve segments treated with (**A**) saline control or (**B**) Btc recombinant protein at 10 days after nerve transection and silicone bridging. Green color indicated NF-H, red color indicated S100β, and blue color indicated nucleus. Boxed areas in (**A** and **B**) were shown in higher magnifications in (**a** and **b**), respectively. (**b**’) was from boxed area of (b). Arrows indicated the regeneration site. Arrowheads indicated Schwann cell-formed cord in the nerve bridge. Scale bars indicated 1000 µm in (**A** and **B**) and indicated 100 µm in (**a** and **b**) and 20 µm in (**b’**). (**C** and **D**) Averaged (**C**) length of regeneration nerve fibers and (**D**) migrating distance of Schwann cells summarized from three experiments. Statistical evaluation was performed using t-test. *p-value < 0.05 versus saline control

Besides sciatic nerve crush, another group of animals were subjected to a 6 mm nerve transection, a more severe injury. A silicone tube injected with Btc recombinant protein or saline control was used to bridge rat sciatic nerve gap. A certain level of Schwann cell migration and axon elongation were observed in both groups at 10 days after surgery (Fig. 6A, B). But rats treated with silicone tubes injected with Btc recombinant protein seemed to possess larger amount of Schwann cells and relatively well-formed Schwann cell-cord in the nerve bridge (Fig. 6B, b, b’) compared to rats receiving saline treatment (Fig. 6A, a). The regeneration conditions of other rats treated with saline control or Btc recombinant protein were shown in Additional file 1. Summarized data showed that the length of regeneration nerve fibers and the migrating distance of Schwann cells were longer in Btc-treated rats (Fig. 6C, D). These observations indicate that Btc encourage Schwann cell migration and axon elongation after peripheral nerve crush injury.

## Discussion

In consideration of the increasing cases of peripheral nerve injury and the inherent disadvantage of autologous nerve grafting, tissue engineered nerve grafts have been more widely applied to treat peripheral nerve injury (Gu et al. 2014). Multiple strategies, such as the modulation of the components/topographic structures of neural scaffolds and the incorporation of stem cells have been made to optimize tissue engineered nerve grafts (Yi et al. 2019b,^ 2020^). Emerging studies demonstrated that growth factors play critical roles during peripheral nerve regeneration (Onger et al. 2017; Li et al. 2020). Therefore, identifying novel growth factors that promote nerve regeneration will further contribute to the construction of growth factor-combined tissue engineered nerve grafts and be beneficial to the treatment of peripheral nerve injury.

A series of growth factors have been discovered to be dysregulated after peripheral nerve injury (Zhang et al. 2019). The mRNA expression of *Btc*, among these dysregulated growth factors, has been identified to be kept up-regulated in the injured nerve stumps at all tested time points (1, 4, 7, and 14 days after nerve injury), implying the potential effect of Btc on Schwann cell phenotype modulation and peripheral nerve regeneration.

Therefore, in the current study, we investigated the biological functions of Btc on peripheral nerve regeneration by using cultured Schwann cells and sciatic nerve injury models. Observations from both Transwell migration assay and wound healing assay showed that *Btc* siRNA transfection and Btc recombinant protein application decreased and increased the spontaneous migration rate of Schwann cells, respectively. Considering that the migration of Schwann cells towards the injured site following peripheral nerve injury is of great significance for success nerve regeneration, the examination of the migration of *Btc* suppressed or overexpressed Schwann cells to a damage signal is meaningful. However, nerve injury-induced triggering signals remain largely undetermined, making it difficult to mimick the injury response in vitro. On the other hand, consistent with in vitro observations, in vivo study showed that the direct application of Btc recombinant protein would accelerate Schwann cell migration, suggesting the essential role of Btc on Schwann cell migration. These observations, together with a previous finding which demonstrated that Btc stimulate the proliferation of myelinating Schwann cells and induce myelin formation (Vallieres et al. 2017), reveal the comprehensive effect of Btc on essential regenerative phenotypes of Schwann cells, i.e., proliferation, migration, and myelination.

Schwann cells not only generate a permissive microenviroment for peripheral nerve regeneration and guide regenerating axonal sprout via forming regeneration tracks, but also provide indispensable supports for neuron survival and growth core formation via producing and secreting trophic factors (Jessen and Mirsky 2016; Mahar and Cavalli 2018; Allodi et al. 2012; Min et al. 2021). For example, it has been demonstrated that c-Jun-gene-modulated-Schwann cells produce many trophic factors and facilitate Schwann cell migration as well as neurite outgrowth (Huang et al. 2015). Our current study reveal that Schwann cells produce and secret Btc to their supernatants and Schwann cell-secreted Btc can influence neuron behaviors and promote neurite outgrowth. Therefore, Btc not only support Schwann cell migration in an autocrine way, but also encourage neurite outgrowth in a autocrine/paracrine way. In vivo observations of the application of Btc at the injury site further demonstrated the promoting roles of Btc on axon growth and elongation after nerve injury using both a mild crush injury model and a more severe transection injury model. Notably, previously, our lab mates immobilized nerve growth factor with biomaterials and found that nerve growth factor-incorporated bio-scaffolds advance nerve regeneration through affecting both Schwann cells and neurons (Li et al. 2017; Yang et al. 2011). Therefore, in future studies, Btc protein or Btc-over expressing Schwann cells may be combined with neural scaffolds to generate Btc-based tissue engineering nerve grafts for the treatment of peripheral nerve regeneration.

## Conclusions

In summary, our current study illuminates that Btc, an elevated growth factors after peripheral nerve injury, can enhance Schwann cell migration, axon elongation, and nerve regeneration. Our study reveals Btc as a regenerative neurotrophic factor, expands the understanding of the involvement of growth factors during peripheral nerve regeneration, and proposes the potential usage of Btc in the treatment of severe peripheral nerve injury.

## Supplementary Information

**Additional file 1: Figure S1**. The immunofluorescence images of rat sciatic nerve segments of the other two rats treated with saline control and the other two rats treated with Btc recombinant protein at 10 days after nerve transection and silicone bridging. Green color indicated NF-H, red color indicated S100β, and blue color indicated nucleus. Arrows indicated the regeneration site. Scale bars indicated 1000 µm.

## Data Availability

Not applicable.
