# Supplementary figures and images for: Betacellulin regulates peripheral nerve regeneration by affecting Schwann cell migration and axon elongation
Source: Mol Med. 2021 Mar 25;27:27. doi: 10.1186/s10020-021-00292-5 (PMC8015203; doi:10.1186/s10020-021-00292-5)

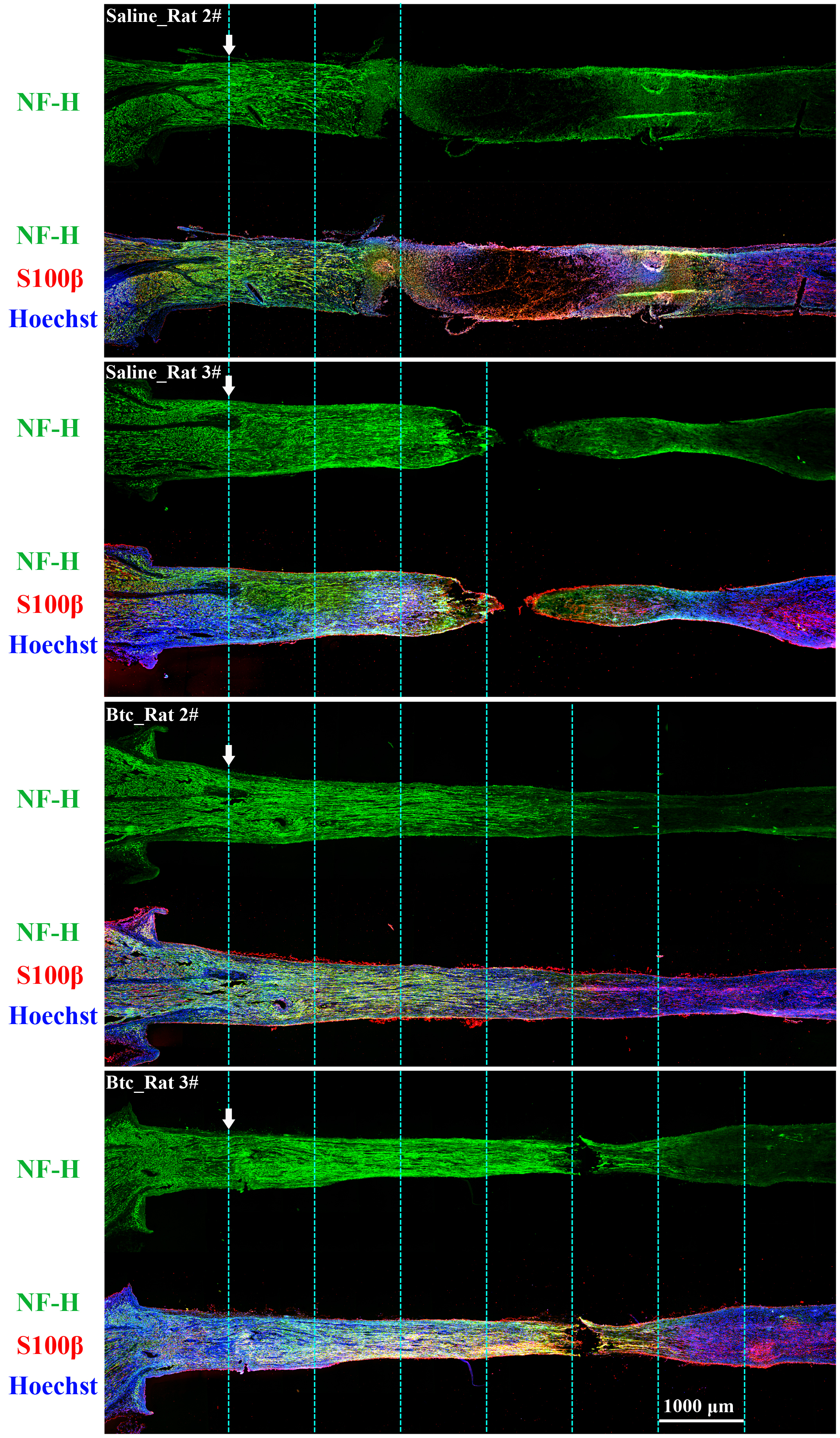

Supplement: Supplementary file 1 — Additional file 1: Figure S1. The immunofluorescence images of rat sciatic nerve segments of the other two rats treated with saline control and the other two rats treated with Btc recombinant protein at 10 days after nerve transection and silicone bridging. Green color indicated NF-H, red color indicated S100β, and blue color indicated nucleus. Arrows indicated the regeneration site. Scale bars indicated 1000 µm. [file 10020_2021_292_MOESM1_ESM.jpg]
